# Molecular typing of *Cyclospora cayetanensis* in produce and clinical samples using targeted enrichment of complete mitochondrial genomes and next-generation sequencing

**DOI:** 10.1186/s13071-020-3997-3

**Published:** 2020-03-06

**Authors:** Hediye Nese Cinar, Gopal Gopinath, Helen R. Murphy, Sonia Almeria, Mauricio Durigan, Dajung Choi, AhYoung Jang, Eunje Kim, RaeYoung Kim, Seonju Choi, Jeongu Lee, Yurim Shin, Jieon Lee, Yvonne Qvarnstrom, Theresa K. Benedict, Henry S. Bishop, Alexandre da Silva

**Affiliations:** 1 Center for Food Safety and Applied Nutrition, U.S. Food and Drug Administration, Laurel, MD USA; 2 Division of Parasitic Diseases and Malaria, Center for Global Health, Centers for Disease Control and Prevention, Atlanta, GA USA

**Keywords:** *Cyclospora cayetanensis*, Mitochondria genome, Genotyping, Next-generation sequencing

## Abstract

**Background:**

Outbreaks of cyclosporiasis, a diarrheal illness caused by *Cyclospora cayetanensis*, have been a public health issue in the USA since the mid 1990’s. In 2018, 2299 domestically acquired cases of cyclosporiasis were reported in the USA as a result of multiple large outbreaks linked to different fresh produce commodities. Outbreak investigations are hindered by the absence of standardized molecular epidemiological tools for *C. cayetanensis*. For other apicomplexan coccidian parasites, multicopy organellar DNA such as mitochondrial genomes have been used for detection and molecular typing.

**Methods:**

We developed a workflow to obtain complete mitochondrial genome sequences from cilantro samples and clinical samples for typing of *C. cayetanensis* isolates. The 6.3 kb long *C. cayetanensis* mitochondrial genome was amplified by PCR in four overlapping amplicons from genomic DNA extracted from cilantro, seeded with oocysts, and from stool samples positive for *C. cayetanensis* by diagnostic methods. DNA sequence libraries of pooled amplicons were prepared and sequenced *via* next-generation sequencing (NGS). Sequence reads were assembled using a custom bioinformatics pipeline.

**Results:**

This approach allowed us to sequence complete mitochondrial genomes from the samples studied. Sequence alterations, such as single nucleotide polymorphism (SNP) profiles and insertion and deletions (InDels), in mitochondrial genomes of 24 stool samples from patients with cyclosporiasis diagnosed in 2014, exhibited discriminatory power. The cluster dendrogram that was created based on distance matrices of the complete mitochondrial genome sequences, indicated distinct strain-level diversity among the 2014 *C. cayetanensis* outbreak isolates analyzed in this study.

**Conclusions:**

Our results suggest that genomic analyses of mitochondrial genome sequences may help to link outbreak cases to the source.
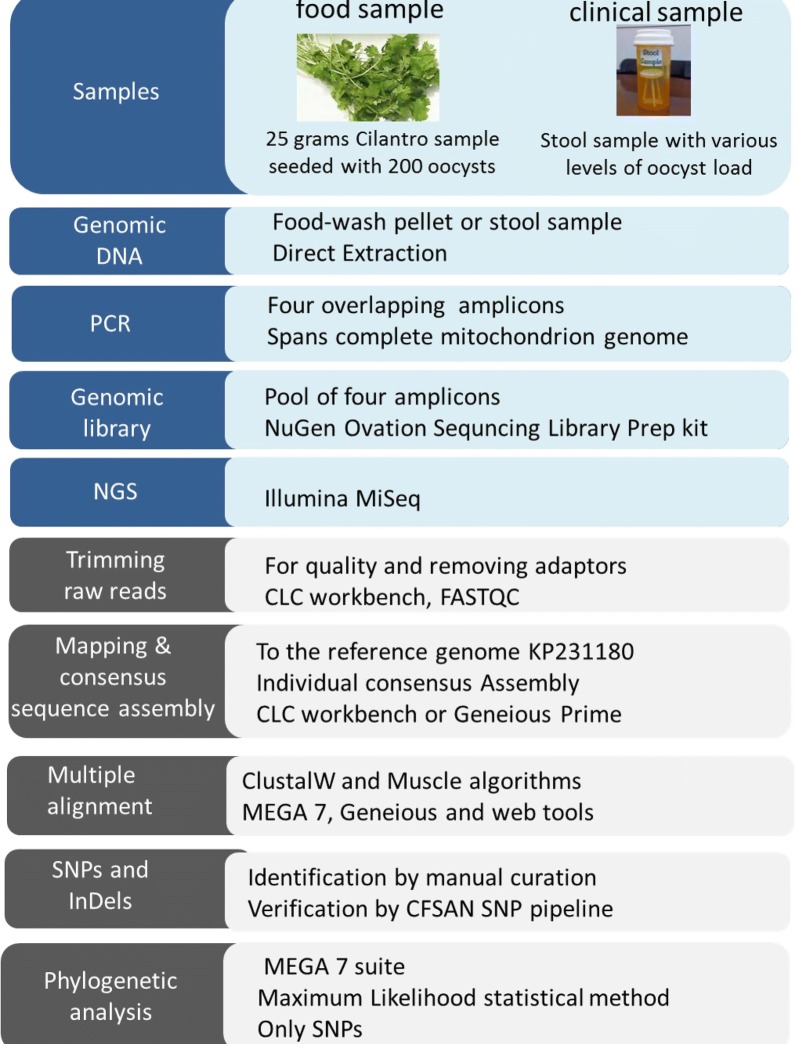

## Background

*Cyclospora cayetanensis* is an apicomplexan protozoan parasite that causes the food- and water-borne diarrheal disease cyclosporiasis worldwide, most commonly in tropical and subtropical regions [1–4]. Large cyclosporiasis outbreaks have been reported in the USA, since the mid-1990’s linked to various types of imported fresh produce (e.g. basil, cilantro, mesclun lettuce, raspberries and snow peas) [5, 6]. Between May and August of 2018, there were multiple cyclosporiasis outbreaks that affected 2299 persons in 33 states of the USA [7] associated with the consumption of different fresh produce items. *Cyclospora cayetanensis* was detected in produce harvested in the USA for the first time in 2018 using a novel FDA method validated for the detection of the parasite in fresh produce [8–10]. A major constraint in outbreak investigations is the lack of molecular epidemiology tools that would be useful in linking patients to the sources of infection.

Until recently, only a scarce amount of *C. cayetanensis* genome sequence information was available due to limited accessibility of oocysts for experimental use. Due to the inability to culture this organism, it has been challenging to generate sufficient genomic DNA from clinical samples for further analysis [5, 11, 12]. Progress on the development of molecular epidemiologic tools to link cases with sources of infection has been hindered by the limited DNA sequence information. With the recent advances in sequencing technologies such as next-generation sequencing (NGS) and availability of efficient genome assembly programs, whole genome assemblies, complete mitochondrial and apicoplast genomes of *C. cayetanensis* have become available [11–18]. NGS is the preferred technology for generation of high-throughput high-resolution data in streamlined fashion for clinical diagnostics, forensic science, and public health research. For example, the FDA’s GenomeTrakr represents a pioneering effort as an open-source whole-genome sequencing network of state, federal, international and commercial partners, for use in characterization of food-borne outbreak pathogens and tracing these back to the source commodity [19]. Although whole genome sequencing of bacterial species such as *Listeria*, *Salmonella* and *E. coli* is becoming a mainstream approach in public health and food safety research, targeted NGS approaches are needed for organisms with larger genomes, and/or for unculturable organisms.

Multicopy mitochondrial genomes have been used for detection and molecular typing in other apicomplexan parasites [20]. Mitochondrial genomes of most apicomplexan species are linear, 6–8 kb in size, with distinct structural variations [21]. The *C. cayetanensis* mitochondrial genome is organized as a concatemeric linear 6.3 kb molecule, which is closely related to the mitochondrial genomes of *Eimeria* species [11]. It contains three protein-coding genes, cytochrome *b* (*cytb*), cytochrome *c* oxidase subunit 1 (*cox*1) and cytochrome *c* oxidase subunit 3 (*cox*3), in addition to 14 large subunit (*LSU*) and nine small subunit (*SSU*) fragmented rRNA genes [11].

**Fig. 1:**
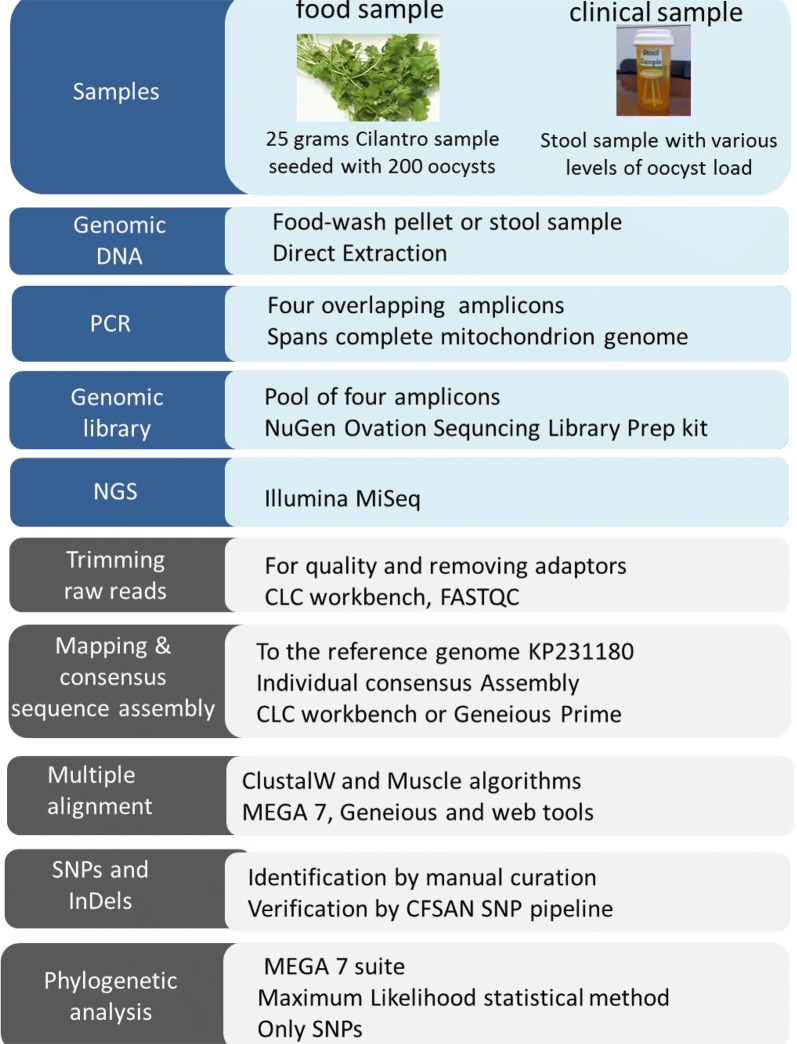
Workflow to generate and analyze mitochondrial genome assemblies from food and clinical isolates of *C. cayetanensis.* Wet-laboratory procedures are shown in blue and bioinformatics analysis steps are in grey

Here we report an experimental workflow that starts with a laboratory component followed by bioinformatic analytical steps, to obtain whole mitochondrial genome sequences from *C. cayetanensis* samples. The steps of our method from the sample DNA preparation to the identification of underlying genetic variation are described in Fig. 1. To implement the method, we first amplified the mitochondrial genome of *C. cayetanensis* in four partially overlapping PCR amplicons from the extracted genomic DNA. DNA sequence libraries of pooled amplicons were used to generate NGS datasets. Using established bioinformatics tools, the raw sequence reads were assembled to generate *de novo* mitochondrial genome assemblies. A detailed comparative analysis of these genomic datasets with the reference mitochondrial genome KP231180 was carried out to identify potential genomic markers such as single nucleotide polymorphisms (SNP) and insertion-deletions (InDels) that could potentially be used for detection and sub-typing. SNP profiles from stool samples from cyclosporiasis cases exhibited discriminatory power. With this approach, we were able to sequence complete mitochondrial genomes not only from patient stool samples, but also from cilantro samples, spiked with *C. cayetanensis* oocysts to simulate contaminated food. Our results suggest that genomic analyses of mitochondrial genomes may reveal intraspecies diversity of *C. cayetanensis* that could be applied to link outbreak cases to sources of infection.

## Methods

### Sources of *C. cayetanensis* samples

Twenty-four stool samples used in this study had been collected in Zn-PVA, TotalFix, AlphaTec, Cu-PVA or Carey Blair medium and submitted to Centers for Disease Control and Prevention (CDC) for either reference diagnosis or as part of outbreak investigations. One stool sample positive for *C. cayetanensis*, labeled C5, originated from Nepal and was generously supplied by Professor Jeevan Sherchand, (Microbiology and Public Health Research Laboratory at Tribhuvan, University Teaching Hospital in Kathmandu, Nepal) and Ynes Ortega (The University of Georgia in Athens, Georgia, USA).

### Generation of oocyst spiked food samples

We obtained *C. cayetanensis* oocysts from a known clinical sample C5 by a purification method described previously [11, 17]. Briefly, *C. cayetanensis* oocysts were recovered from sieved fecal samples by differential sucrose and cesium chloride gradient centrifugations. *Cyclospora cayetanensis* oocysts were counted using a haemocytometer and fluorescent microscopy using a Zeiss Axio Imager D1 microscope (Zeiss, Oberkochen, Germany) with an HBO mercury short arc lamp and a UV filter (350 nm excitation and 450 nm emission). Then, known numbers of purified oocysts were added onto cilantro. DNA was extracted from this spiked cilantro sample as described in Murphy et al. [9]. Briefly, 25 g of cilantro sample were spiked with 200 oocysts, incubated at 4 °C for 48 h and washed with 0.1% Alconox (Alconox Inc., NY, USA). After centrifugation of the produce wash, debris pellets were collected, and DNA extracted using the FastDNA SPIN Kit for soil (MP Bio, Santa Ana, California).

### Microscopical and molecular analysis of stool specimens

Stool specimens from CDC were analyzed for the presence of *C. cayetanensis* by UV fluorescence microscopy. DNA was extracted directly from these samples and analyzed using a real-time PCR (qPCR) method targeting the *C. cayetanensis 18S* rRNA gene following protocols and conditions described elsewhere [22, 23]. DNA from samples that were positive for *C. cayetanensis* by microscopy and/or qPCR was selected for this study. Samples were classified in different categories of positivity (high, medium and low) for *C. cayetanensis* based on the oocyst load and qPCR cycle threshold (Cq) values. Samples that had more than 10 oocysts in 1 to 10 microcopy fields and had qPCR Cqs below 29 were classified as having high positivity, samples that had between 1–10 oocysts in 10 microscopic fields with qPCR Cqs between 30–33 were classified as medium positivity, and samples that had ≤ 1 oocyst within 10 microscopic fields and had qPCR Cqs above 33 were classified as low positivity.

### PCR amplification of the whole *C. cayetanensis* mitochondrial genome

PCR reactions were performed using the Platinum PCR SuperMix High Fidelity Kit (Invitrogen, Grant Island, NY, USA) according to the manufacturer’s instructions on an AB 2720 thermal cycler (Applied Biosystems, Waltham, Massachusetts, USA). DNA extracted from stool samples and spiked cilantro samples was used as a template. The PCR primers (Table 1) were designed to amplify the whole mitochondrial genome (6.3 kb) in four overlapping amplicons. PCR was performed in a 50 µl volume reaction containing 2 µl of template DNA, 1 µl each primer (final concentration 400 nM each) and 46 µl Platinum PCR SuperMix (Invitrogen). PCR conditions for amplification of the four fragments were as follows: amplicon 1 (95 °C for 3 min followed by 40 cycles of denaturation at 95 °C for 30 s, annealing at 50 °C for 30 s and extension at 65 °C for 3 min, with a final extension step at 65 °C for 10 min); amplicon 2 and 3 (95 °C for 3 min followed by 40 cycles of denaturation at 95 °C for 30 s, annealing at 55 °C for 30 s and extension at 65 °C for 3 min, with a final extension step at 65 °C for 10 min); amplicon 4 (95 °C for 3 min followed by 40 cycles of denaturation at 95 °C for 30 s, annealing at 52 °C for 30 s and extension at 65 °C for 3 min, with a final extension step at 65 °C for 10 min).

**Table 1:** Primer sequences used for PCR amplification of *C. cayetanensis* complete mitochondrial genome

Primer	Sequence (5′–3′)
1F (2F-begin2)	ATGTTTTTAATGTCTCAAGTGAGATCTCAT
1R (Fragment 1-R-2)	GGTTTGCAGCAGTTAGAATACTAGAATTAG
2F (Fragment 2-F-1)	GTTGGAGCTCAATTACCTCAAGAAGTATTC
2R (3Rb)	TTTGTATGGATTTCACGGTCAACTC
3F (F3-Frag3-of-4)	GTAACTCCGCTCTAGATGTTGCTTTACACG
3R (R2-Frag3-of-4)	CCTCAGTTGGACTTACTAGGGTGGAGTCTG
4F (Fragment1-F-4)	GGTTTCATCAATTTGTTTAGGTGTTATTAG
4R (End6R)	CACATGATGCT CAGTAGCATGTAGG

### Next-generation sequencing of PCR amplicons

For each sample, PCR products from the four amplicons were pooled into one tube and quantified using a Qubit 1.0 and the Qubit dsDNA BR Assay Kit (Life Technologies, Grand Island, NY, USA). One hundred ng of total DNA was used to prepare NGS libraries using Ovation Ultralow System V1 Library Preparation Kit (NuGEN, San Carlos, CA, USA). Approximately 10–16 pmol of each library was paired-end sequenced on the MiSeq platform (Illumina) following the manufacturerʼs manual.

### Generation of mitochondrial genome assemblies

The CLC Genome Workbench Toolkit 9.0 (Qiagen, Hilden, Germany) was used for trimming the raw NGS reads based on quality and to remove the adaptor sequences as recommended. Assembly and analysis of the reads were carried out in multiple steps following a combination of methods reported earlier [11, 18]. Briefly, the reads were first mapped to the reference genome KP23180 on Geneious 9. A reference-guided consensus sequence ‘consensus assembly’ was then exported as a fasta sequence file for each dataset that corresponded to the mitochondrial genome assembly. In parallel, CLC workbench 9.0 was used to generate a *de novo* assembly for each sample. First, the reads mapping to KP231180 were collected as ‘mapped reads’ which were trimmed to remove adaptors and low-quality reads. *De novo* assemblies were generated from mapped, trimmed reads using default conditions. Manual curation of each *de novo* assembly was carried out to re-shuffle the genomic start-stop positions to align with that of the reference mitochondrial genome KP231180. Congruency of the reference and sample genome assemblies was obtained by re-aligning terminal fragments and smaller contigs and manually ‘stitching’ them together when necessary to obtain a single-molecule assembly [18]. Each curated assembly was verified for integrity by re-mapping to KP231180. Twenty-four of this curated set of 25 *de novo* assemblies were used for downstream bioinformatic analysis. NGS datasets from spiked cilantro sample were processed as above and compared with the mitochondrial genome of the source (*C. cayetanensis* C5 isolate from Nepal; GenBank: MG831586) and the reference genome KP231180.

### Phylogenetic analysis of mitochondrial genomes

Twenty-four genome assemblies obtained from stool samples (this study) and three genomes (MG831586, MG831587 and MG831588) reported by Gopinath et al. [18] were aligned with KP231180 using the Muscle algorithm implemented in MEGA 7 [24] to identify the distribution of 15 bp repeats in the distal repeat region and nucleotide variation profiles spanning the genomes. The respective positions for SNPs and InDels were calculated based on KP231180 sequence. First, SNP positions and InDels were identified in the distal ‘repeat-region’ [18, 25], by manual curation using both programs. To avoid alignment-based errors for detecting InDels, multiple alignment analysis was independently carried out using the same dataset applying ClustalW and Muscle algorithms implemented in Geneious and MEGA software suites, and separately on MAFFT (https://mafft.cbrc.jp/alignment/server) and ClustalW (https://www.ebi.ac.uk/Tools/msa) tools. The mapped reads were analyzed using CFSAN SNP-Pipeline to verify the curated SNPs using the program’s default configuration. The stringent configuration of the pipeline is intolerant to reads with lower read quality, which is essential to maintain high confidence SNP calls during the analysis of genomes from samples implicated in food contamination and associated clinical samples during outbreak events [25]. SNPs and InDels in the reads mapped to KP231180 from each sample were verified independently by visualization in Geneious to confirm the observations from genome alignments and SNP calling software. All base positions indicated in this report are based on the reference genome KP231180 unless specifically noted.

For clustering, a pairwise distance matrix was computed using Maximum Composite Likelihood method implemented in MEGA7. The distance matrix used for cluster analysis is available in the Additional file 1: Table S1. An optimal dendrogram illustrating clusters of isolates each sharing similar genotype was generated using Maximum Likelihood statistical method and visualized as a circular tree. The pairwise-distance was tested by re-sampling the cluster generation 1000 times and independently with in-built clustering tools in Geneious (data not shown) for validating the resulting clusters.

### Submission of genome datasets to NCBI

Metadata, sequencing reads and genome assemblies from the 24 USA samples were submitted to Bioproject under CycloTrakr Database (accession PRJNA357477) at NCBI (www.ncbi.nih.gov) (Additional file 2: Table S2).

## Results

### PCR amplification of complete mitochondrial genomes from fresh produce and stool samples

**Fig. 2:**
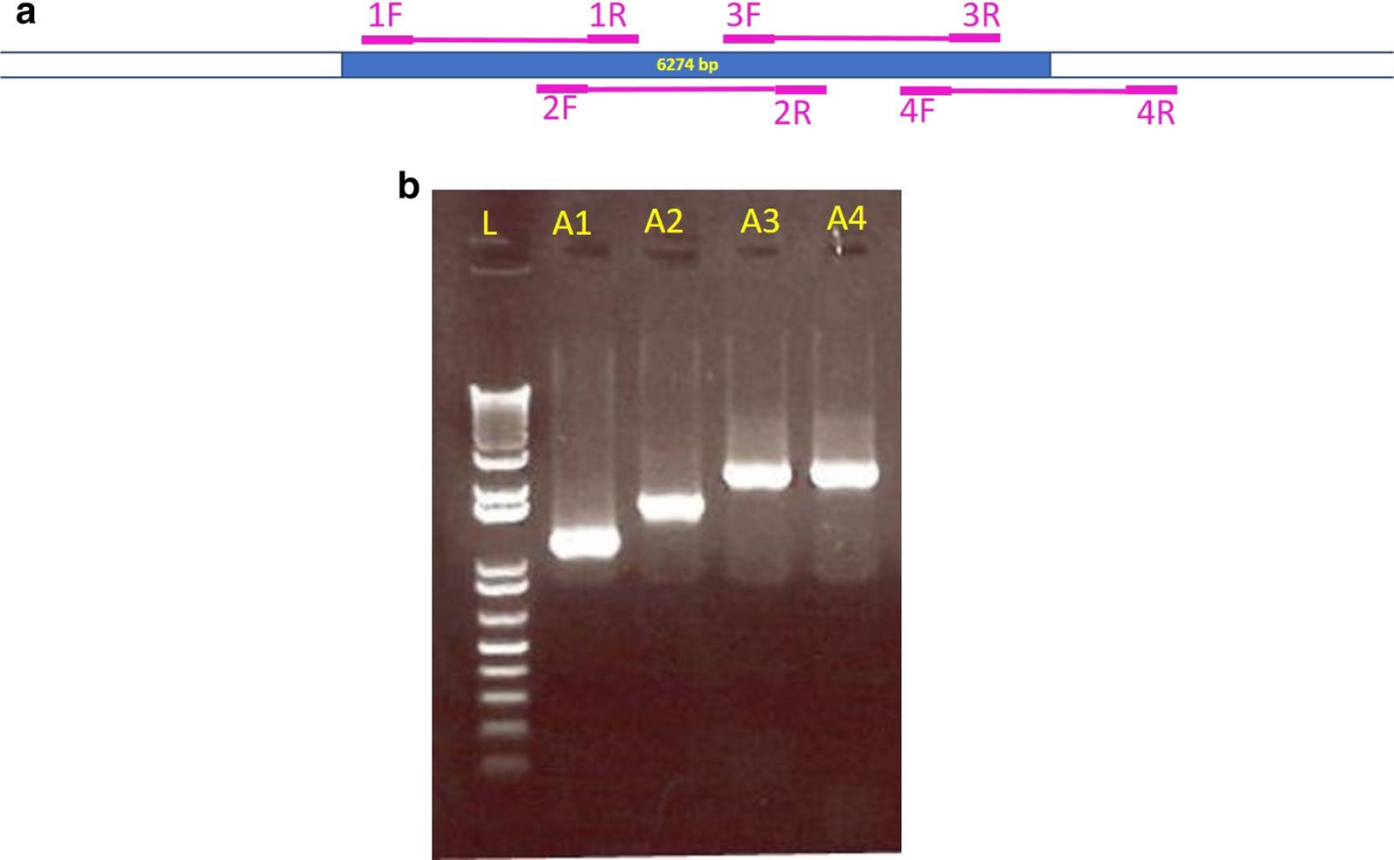
Amplification of complete *C. cayetanensis* mitochondrial genome in four overlapping amplicons. **a** Schematic representation of mitochondrial genome in concatemeric structure, with complete genome (in blue) flanked with partial genomes (in white). Relative places of primers and resulting amplicons are shown in pink. **b** Agarose gel electrophoresis image of amplicon DNA run with 1 kb plus ladder

To identify and type the *C. cayetanensis* isolates based on the sequence diversity, we amplified the 6.3 kb long *C. cayetanensis* mitochondrial genome from genomic DNA extracted from cilantro spiked with oocysts and from stool samples (see “Methods”). The definitions of the four sets of PCR primers and the experimental results of four overlapping fragments are described in Tables 1, 2 and Fig. 2. Due to the AT-rich nature of the *C. cayetanensis* mitochondrial genome (67% AT), primers 25 bp to 30 bp in length were used to achieve melting temperatures (Tm) from 55 °C to 62 °C, to increase amplification efficiency. A lower extension temperature (65 °C) was used to prevent DNA melting, which may hinder extension of AT-rich sequences [26].

**Table 2:** Primer set and PCR fragment information

Amplicon	Forward primer	Reverse primer	Amplicon size (bp)
A1	1F (2F-Begin2)	1R (Fragment 1-R-2)	1160
A2	2F (Fragment 2-F-1)	2R (3Rb)	1609
A3	3F (F3-Frag3-of-4)	3R (R2-Frag3-of-4)	2219
A4	4F (Fragment1-F-4)	4R (End6R)	2652

Stool samples were classified in levels of positivity for *C. cayetanensis* based on the oocyst load evaluated *via* microscopy and qPCR Cq values (see “Methods”). Total DNA extracted directly from stool samples with various oocyst loads was used to generate complete mitochondrial genomes of *C. cayetanensis* (Table 3). We were able to obtain complete mitochondrial genomes even from the stool samples with low positivity (≤ 1 oocyst within 10 microscopic fields and qPCR Cq values above 33). In addition, we were able to amplify the complete mitochondrial genome of *C. cayetanensis* from a produce sample seeded with 200 oocysts using this method, exhibiting the viability of the method in food samples as a proof of principle. Our results demonstrate the feasibility of using any type of sample that could be gathered either from patients or food in an outbreak to assess the genetic variation of *C. cayetanensis* as the disease-causing and/or contaminating agent.

**Table 3:** Stool specimens used in this study

Sample name	State	Year	Sample	Preservative	Positivity for *C. cayetanensis*
TX_14_CL_17	TX	2014	Stool	Zn-PVA	High
TX_14_CL_21	TX	2014	Stool	Zn-PVA	High
TX_14_CL_6	TX	2014	Stool	Zn-PVA	Low
TX_14_CL_22	TX	2014	Stool	Zn-PVA	High
TX_14_CL_23	TX	2014	Stool	Total fix	High
MA_14_CL_2	MA	2014	Stool	Total fix	Medium
ME_14_CL_24	ME	2014	Stool	Total fix	Medium
ME_14_CL_25	ME	2014	Stool	Total fix	Medium
TX_14_CL_19	TX	2014	Stool	Zn-PVA	High
TX_14_CL_18	TX	2014	Stool	Zn-PVA	Medium
TX_14_CL_15	TX	2014	Stool	Zn-PVA	Low
TX_14_CL_14	TX	2014	Stool	Zn-PVA	Medium
TX_14_CL_13	TX	2014	Stool	Zn-PVA	Medium
MA_14_CL_11	MA	2014	Stool	Total fix	Medium
TX_14_CL_16	TX	2014	Stool	Zn-PVA	Medium
MT_14_CL_5	MT	2014	Stool	AlphaTec	Low
OH_14_CL_1	OH	2014	Stool	Cu-PVA	High
MA_14_CL_3	MA	2014	Stool	Total fix	Medium
MA_14_CL_4	MA	2014	Stool	Total fix	Medium
MA_14_CL_10	MA	2014	Stool	Total fix	Medium
MA_14_CL_7	MA	2014	Stool	Total fix	Low
MA_14_CL_9	MA	2014	Stool	Total fix	Medium
SC_14_CL_26	SC	2014	Stool	Carey Blair	NPF
TX_14_CL_20	TX	2014	Stool	Zn-PVA	Low
F200	Nepal	2014	Food^a^	na	na

^a^Cilantro sample seeded with 200 *C. cayetanensis* oocyts

*Abbreviations*: na, not available; npf; no parasite found

### Next-generation sequencing and assembly of mitochondrial genomes

For each sample, NGS reads resulting from libraries of pooled amplicons were mapped to mitochondrial reference genome KP231180 to gather mitochondrion specific reads. We achieved very high average read coverage; between 8961 X and 92691 X, which provided high confidence for calling SNPs and other sequence variations (Additional file 2: Table S2). The ratios of the reads from different samples mapping to the reference sequence varied within the range of 66–99% (Additional file 2: Table S3), presumably due to the inherent differences in the PCR amplification and varying read qualities of the associated NGS datasets. Mapping to the reference sequence led to a consensus assembly representing the entire *C. cayetanensis* mitochondrial genome sequence specific for each sample. Additionally, the mapped reads were extracted and assembled individually to generate 24 *de novo* assemblies for cluster analysis of mitochondrial genomes. The consensus assemblies were manually curated using the visualized mapping of reads and compared with the *de novo* assemblies to remove anomalous base-calling. This resulted in identical sequences in both consensus and *de novo* assemblies for each sample. In the workflow outlined in Fig. 1, consensus assembly was used as the starting point for subsequent multiple alignment.

### Identification of SNPs and InDels in the mitochondrial genomes of different samples

**Fig. 3:**
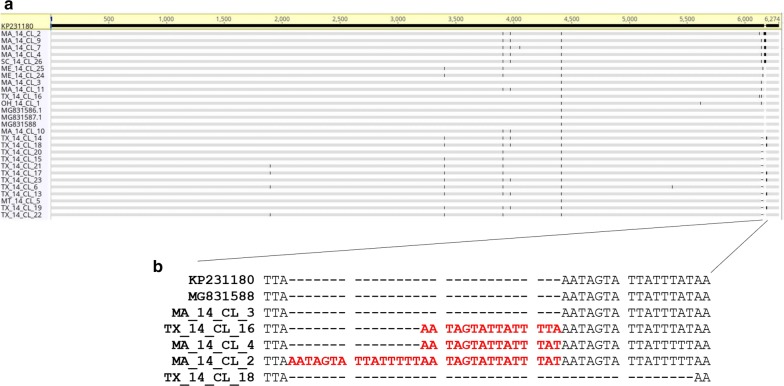
Sequence variations identified in the present study. **a** Single nucleotide polymorphisms and InDels shown in Geneious visualization panel. **b** Distal repeat region sequence alignment

We identified 12 single nucleotide polymorphisms (SNPs) across the mitochondrial genome (Fig. 3a, Table 4) by manually curating the multiple alignment of 24 genomes from this study and three from earlier work [18] with the reference genome KP231180. These 12 ‘hot spot’ positions were enumerated based their positions on the KP231180 sequence. Curation of multiple alignment data by different methods as described earlier identified insertions and deletions (InDels) in the terminal repeat region. The nucleotide polymorphisms observed in the 12 ‘allelic hotspots’ and the InDels were consolidated as a Variome matrix of mitochondrial genome (Additional file 2: Table S4). All 24 genomes from this study contained a SNP at base 4415 of the reference genome. One sample, MT_14_CL_5 was identical to the published Nepalese samples except for a deletion. Twenty out of 24 query samples contained at least one insertion (‘I’) or a deletion (‘D’) (Additional file 2: Table S4). CFSAN-SNP pipeline confirmed the 12 SNPs among 22 of 24 samples (Table 4). The stringent configuration of the pipeline is intolerant to reads with lower read quality, which is essential to maintain high confidence SNP calls during the analysis of genomes from samples implicated in food contamination and associated clinical samples during outbreak events [27]. Variations in the occurrence of 15-bp repeats in the distal region were observed (terminal end, Fig. 3b) in the samples used in this study. The variations in this region, initially observed in MEGA 7 alignment and Geneious mapping, were verified by multiple alignments with MAFFT (Fig. 3b). An insertion of ‘AAT AGT ATT ATT TA’ in sample TX_14_CL_16, ‘AAT AGT ATT ATT TAT’ in MA_14_CL_4, MA_14_CL_7, and ME_14_CL_24 and a unique insertion of tandem repeat ‘AAT AGT ATT ATT TTT AAT AGT ATT ATT TAT’ in sample MA_14_CL_2 (Fig. 3b). A deletion of 14 bases from 6156 to 6170 consisting of ‘AAT AGT ATT ATT TAT’ was observed in 50% of the samples tested (Fig. 3a). All these observations point to hypervariability in the sequences of this ‘distal region’. *Cyclospora cayetanensis* mitochondrial genome sequences obtained from cilantro samples seeded with the Nepalese isolate C5 (NCBI BioSample SAMN04870148) were found to be identical to mitochondrial genome MG831586 (data not shown), which was originally sequenced directly from oocysts from the same stool sample [18]. All the steps from sample DNA preparation to the identification of SNPs and InDels were consolidated to a workflow for routine (Fig. 1). This workflow could be implemented as part of any bioinformatic pipeline for routine use. It conveniently uses the mapping of reads for generating ‘consensus assembly’ verified by *de novo* assemblies from the read datasets, and SNP and InDel calling independently confirmed by multiple alignment and SNP calling as shown above.

**Table 4:** Allelic hot spots identified in the reference genome from this study

#	Allele	Variants	Reference genome position
1	G	A	1898
2	T	G	3404
3	A	C	3910
4	T	G	3973
5	T	G	4055
6	C	A	4415
7	C	T	5371
8	A	T	5616
9	A	T	6126
10	A	T	6141
11	T	A	6170
12	T	A	6172

### Phylogenetic analysis of *C. cayetanensis* mitochondrial genomes

**Fig. 4:**
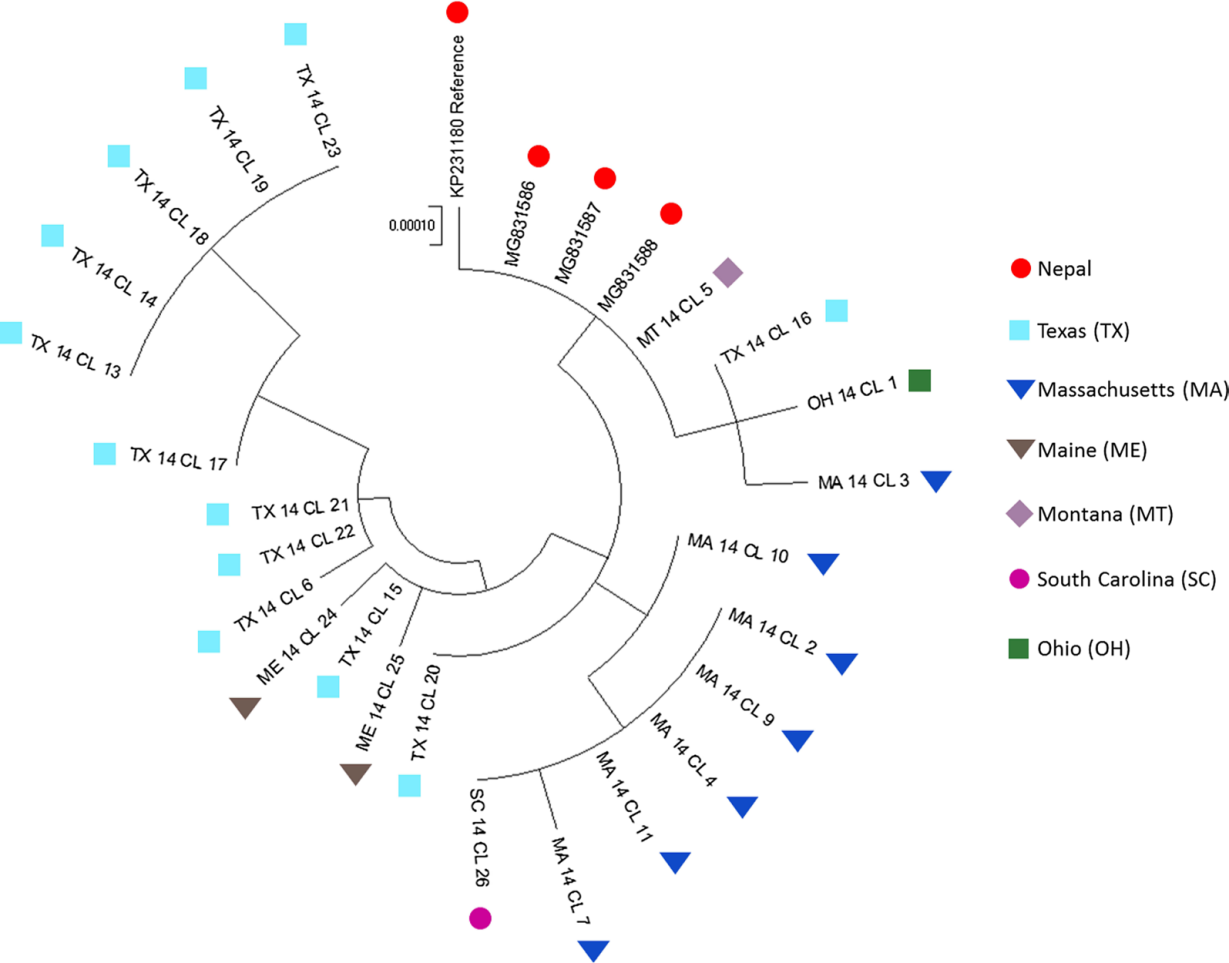
Cluster analysis of *C. cayetanensis* mitochondrial genomes in samples from different geographical areas. Cluster dendrogram was generated based on mitochondrial genome sequence data from 24 samples from 2014 sample collection and tree published *C. cayetanensis* mitochondrial genomes. The specimens are color coded according to their collection locations. Reference genome KP231180 and previously published *C. cayetanensis* mitochondrial genomes MG831586, MG831587 and MG831588 are sequenced from specimens originated from Nepal. The circular dendrogram was constructed in MEGA 7. The initial distance matrix was computed using the Maximum Composite Likelihood method. The option ‘complete deletion’ was chosen which deleted the bases across all genomes even if a single gap. There were a total of 6258 positions in the final dataset

A phylogenetic analysis of 28 mitochondrial genomes including the reference genome KP231180 resulted in a dendrogram (Fig. 4), which illustrates the grouping of the samples from different geographical areas of the USA and Nepal based on their mitochondrial genome profiles. The 28 samples from the different USA states and Nepal fell into several distinct groups. We observed a trend where samples originating from the same geographical locale are clustering together (Fig. 4). The samples from outbreaks in geographical areas coded as Montana (MT), Maine (ME), South Carolina (SC) and Ohio (OH) were clustered together with samples originating from other geographical regions, presumably due a lack of similar samples causing low resolving power. In sum, our analysis resulted in an allele-based classification scheme for outbreak samples which may generate new hypotheses regarding the sources of infection.

## Discussion

To facilitate molecular typing in outbreak investigations, we developed a method to generate the sequence diversity information about the *C. cayetanensis* mitochondrial genomes from patient and food samples (Fig. 1). The method has laboratory and bioinformatic components. The laboratory steps of this method include sample DNA preparation, targeted amplification of mitochondrial genomes, and next-generation sequencing of the pooled amplicons. The bioinformatic steps involve the identification of the scope of individual sequence variation in a given set of samples, and the classification of each sample into groups according to the underlying sequence differences. To our knowledge, this is the first report of a molecular typing method for *C. cayetanensis* applied to both clinical stool samples and to produce (cilantro) seeded with a known number of *C. cayetanensis* oocysts.

Historically, only a limited number of outbreak cases of cyclosporiasis have been linked to specific food exposures due to the complex nature of the epidemiological trace back and lack of molecular epidemiology tools. In the cyclosporiasis outbreaks that occurred in 2018, 68% of the laboratory-confirmed domestically acquired cases were not linked to definitive types of food exposure [7]. Hence, obtaining sequence information from clinical and food isolates and developing molecular epidemiology tools to track the source of *C. cayetanensis* contamination in food are of major public health interest. Despite the significant clinical and public health importance of *C. cayetanensis*, genetic information was sparse (limited to ribosomal complex and heat-shock protein sequences) due to technical difficulties and limited accessibility of *C. cayetanensis* samples. Since 2014, FDA and CDC in collaboration with academia developed methods and strategies to sequence *C. cayetanensis* genomes, including complete mitochondrial and apicoplast genomes, and high quality whole genome assemblies using NGS technologies [11–18].

With the advances in NGS technologies, whole genome sequencing (WGS)-based typing has become a preferred approach in clinical and public health microbiology in the recent years [28]. There are several examples of outbreak assessments using WGS typing for the tracking and identification of prokaryotic microorganisms, such as bacteria [29–31]. This is currently not a viable approach for *C. cayetanensis*, an unculturable eukaryotic parasite having a much larger genome (~ 45 Mb) compared to bacteria. Another complicating factor is the degree of variations due to genetic recombination events in subpopulations of this parasite, which may result in high genome heterozygosity and disruption of the multi-locus SNP associations.

Following the generation of *C. cayetanensis* whole genome assemblies, several studies aimed at the development of typing tools have been published. Guo et al. [32] reported the development of a multi-locus sequence typing (MLST) tool based on microsatellite and minisatellite sequences found in the *C. cayetanensis* genome. A total of 34 isolates with complete sequence data at five loci, formed 25 MLST types. The authors reported several challenges such as failure of nested PCR reactions and failure of Sanger sequencing reactions presumably due to mixed *C. cayetanensis* populations, which may lead to unreadable sequences [32]. Another problem reported by the authors was that the geographical clustering pattern of specimens from the same country at one locus did not confirm to patterns at other loci, presumably because of genetic recombination events [32, 33]. In another study, the MLST method was applied to 76 *C. cayetanensis-*positive samples from China. Forty-five specimens were positive for all five *C. cayetanensis* microsatellite loci and 29 MLST types were detected three distinct clusters were defined. The *C. cayetanensis* isolates from China formed two main clusters, and these clusters were significantly different from a third cluster, which was mainly formed by the isolates from the USA, Peru and Nepal. The authors concluded that presence of strong linkage disequilibrium (LD) supports a significant clonal structure in *C. cayetanensis* in China. In a recent study by Hofstetter et al. [34], the authors used the same MLST method to analyze *C. cayetanensis* positive stool samples collected during 1996–2016 from 54 patients in the USA. They reported that the original MLST technique failed to assign a type to a high proportion of specimens due to unreadable sequences and that a revised method based on the two loci with the best sequencing results had better performance but could not reliably differentiate unrelated specimens. In a recent publication, Barratt et al. [35] reported an ensemble of two similarity-based classification algorithms, including a Bayesian and heuristic component to evaluate the relatedness of *C. cayetanensis* infections. The analysis method described in the paper was designed to address the issue of heterozygosity which is stemming from the eukaryotic nature of the organism. This approach requires a set of haplotypes as input and assigns distances based on probabilities to specimen pairs to define their relationships. When this classification algorithm was applied to the sequence data generated from 88 human stool samples containing *C. cayetanensis*, it generated plausible clusters of genetically related infections with some concordance with epidemiologically defined outbreak clusters of cyclosporiasis.

Organelle genomes, such as genomes of the mitochondria and apicoplasts, are particularly informative in tracing population dynamics due to their non-recombining nature [20]. We reported the complete apicoplast genome of *C. cayetanensis*, and performed an intraspecies comparative sequence analysis of genomes from samples collected in Nepal, New York, Texas and Indonesia, and concluded that SNPs and sequence repeats identified in this study may be useful as genetic markers for geo-genomic identification and classification of *C. cayetanensis* [17]. Mitochondrial DNA has been widely used in phylogenetics [36–38], evolutionary biology [38], forensic sciences [39, 40] and anthropology [41] due to its unique attributes, such as maternal inheritance without recombination events [42, 43] and relatively higher mutation rate compared to nuclear DNA [44, 45]. Although it may not be as discriminating as nuclear genome loci, mitochondrial genome analysis may be used to identify groups that descended from a single source and distinguish between populations from diverse geographical regions. Nuclear genome loci, although with its potential for higher discriminatory power, may have limited use in typing due to recombination events during meiosis that could introduce substantial genetic variations within related populations. Therefore, mitochondrial genomes may serve as important tools for outbreak investigations where back-tracking from individual cases to the outbreak source is crucial [40]. Furthermore, the multi-copy nature of mitochondrial genomes in cells provides higher target sequence concentration than nuclear sequences for molecular methods, such as PCR and NGS. Altogether these characteristics make mitochondrial DNA an excellent target for the development of the molecular epidemiology methods for *C. cayetanensis*.

Two recent studies, Guo et al. [25] and Nascimento et al. [46], reported usage of the 300 bp region containing the distal repeat sequences of the mitochondrial genome of *C. cayetanensis* as a target to assess genetic heterogeneity among clinical isolates of this organism. Based on qPCR melt curve, electrophoresis analysis, and DNA sequence analysis of qPCR products, Guo et al. [25] were able to rapidly assess genetic heterogeneity among *C. cayetanensis* isolates but they did not assess typing resolution and source tracking ability of this approach. Nascimento et al. [46] used deep amplicon sequencing to analyze samples linked to case clusters and compared genetic data with epidemiological information obtained during outbreak investigations. Although 14 genotypes of this distal repeat region were identified, it was not sufficient to resolve all outbreak clusters included in the study.

## Conclusions

The present study describes a method to sequence the complete mitochondrial genomes of *C. cayetanensis* to capture informative SNPs (allelic hot spots) and the hypervariable distal repeat region. The approach presented here circumvents the problem of genetic recombination by using sequences from an organellar genome. As far as we are aware, this is the first study highlighting the underlying possible diversity of *C. cayetanensis* subpopulations exclusively based on mitochondrial genome sequence variations. The method was successfully used on stool samples, including those with low positivity, as well as a food matrix (cilantro) spiked with 200 oocysts. The present method performs well in situations where a food commodity is contaminated with one isolate of *C. cayetanensis*. If a food sample is contaminated with more than one isolate, accurate assembly of individual genotypes and determination of the SNPs in each distinct isolate may not be possible using this method. The resolution and the accuracy of identification of nucleotide polymorphisms using this approach will be significantly affected in the presence of more than one source of contamination in any given sample. We propose that the described method could facilitate the public health response to *C. cayetanensis* outbreaks by providing a genomics tool linking *C. cayetanensis* in clinical and food samples during outbreak investigations. Mitochondrial genomes generated by this and similar methods can be uploaded to the publicly accessible *C. cayetanensis* genome database CycloTrakr (on NCBI) within the GenomeTrakr network and thus be available for future epidemiological investigations of food-borne outbreaks of this organism.

## Supplementary information

**Additional file 1: Table S1.** The distance matrix used for cluster analysis.

**Additional file 2: Table S2.** List of mitochondrial genome assemblies from the present study. **Table S3.** Results of mapping and assembly of sequencing reads. **Table S4.** Variome matrix of mitochondria genome with SNP’s and InDels.

## Data Availability

Metadata and genome assemblies from the 24 USA samples were submitted to Bioproject under CycloTrakr Database (accession PRJNA357477) at NCBI. (www.ncbi.nih.gov).

## Abbreviations

bp: base pairs
kb: kilobase
Mb: megabase
UV: ultraviolet
USA: Unites States of America
FDA: US Food and Drug Administration
CDC: Center for Disease Control and Prevention
rRNA: ribosomal RNA

## References

[1] Ortega YR, Sterling CR, Gilman RH, Cama VA, Díaz F (1993). *Cyclospora* species—a new protozoan pathogen of humans. N Engl J Med..

[2] Ortega YR, Gilman RH, Sterling CR (1994). A new coccidian parasite (Apicomplexa: Eimeriidae) from humans. J Parasitol..

[3] Ortega YR, Sanchez R (2010). Update on *Cyclospora cayetanensis*, a food-borne and waterborne parasite. Clin Microbiol Rev..

[4] Sterling CR, Ortega YR (1999). *Cyclospora:* an enigma worth unraveling. Emerg Infect Dis..

[5] Herwaldt BL (2000). *Cyclospora cayetanensis:* a review, focusing on the outbreaks of cyclosporiasis in the 1990s. Clin Infect Dis..

[6] Abanyie F, Harvey RR, Harris JR, Wiegand RE, Gaul L, Desvignes-Kendrick M (2015). 2013 multistate outbreaks of *Cyclospora cayetanensis* infections associated with fresh produce: focus on the Texas investigations. Epidemiol Infect..

[7] Casillas SM, Bennett C, Straily A (2018). Notes from the field: multiple cyclosporiasis outbreaks—United States, 2018. Am J Transplant..

[8] Almeria S, da Silva AJ, Blessington T, Cloyd TC, Cinar HN, Durigan M (2018). Evaluation of the US Food and Drug Administration validated method for detection of *Cyclospora cayetanensis* in high-risk fresh produce matrices and a method modification for a prepared dish. Food Microbiol.

[9] Murphy HR, Lee S, da Silva AJ (2017). Evaluation of an improved US Food and Drug Administration method for the detection of *Cyclospora cayetanensis* in produce using real-time PCR. J Food Prot..

[10] Murphy HR, Cinar HN, Gopinath G, Noe KE, Chatman LD, Miranda NE (2018). Interlaboratory validation of an improved method for detection of *Cyclospora cayetanensis* in produce using a real-time PCR assay. Food Microbiol..

[11] Cinar HN, Gopinath G, Jarvis K, Murphy HR (2015). The complete mitochondrial genome of the foodborne parasitic pathogen *Cyclospora cayetanensis*. PLoS ONE..

[12] Qvarnstrom Y, Wei-Pridgeon Y, Van Roey E, Park S, Srinivasamoorthy G, Nascimento FS (2018). Purification of *Cyclospora cayetanensis* oocysts obtained from human stool specimens for whole genome sequencing. Gut Pathog..

[13] Ogedengbe ME, Qvarnstrom Y, da Silva AJ, Arrowood MJ, Barta JR (2015). A linear mitochondrial genome of *Cyclospora cayetanensis* (Eimeriidae, Eucoccidiorida, Coccidiasina, Apicomplexa) suggests the ancestral start position within mitochondrial genomes of eimeriid coccidia. Int J Parasitol..

[14] Tang K, Guo Y, Zhang L, Rowe LA, Roellig DM, Frace MA (2015). Genetic similarities between *Cyclospora cayetanensis* and cecum-infecting avian *Eimeria* spp. in apicoplast and mitochondrial genomes. Parasites Vectors..

[15] Qvarnstrom Y, Wei-Pridgeon Y, Li W, Nascimento FS, Bishop HS, Herwaldt BL (2015). Draft genome sequences from *Cyclospora cayetanensis* oocysts purified from a human stool sample. Genome Announc..

[16] Liu S, Wang L, Zheng H, Xu Z, Roellig DM, Li N (2016). Comparative genomics reveals *Cyclospora cayetanensis* possesses coccidia-like metabolism and invasion components but unique surface antigens. BMC Genom.

[17] Cinar HN, Qvarnstrom Y, Wei-Pridgeon Y, Li W, Nascimento FS, Arrowood MJ (2016). Comparative sequence analysis of *Cyclospora cayetanensis* apicoplast genomes originating from diverse geographical regions. Parasites Vectors..

[18] Gopinath GR, Cinar HN, Murphy HR, Durigan M, Almeria M, Tall BD (2018). A hybrid reference-guided *de novo* assembly approach for generating *Cyclospora* mitochondrion genomes. Gut Pathog..

[19] Allard MW, Strain E, Melka D, Bunning K, Musser SM, Brown EW (2016). Practical value of food pathogen traceability through building a whole-genome sequencing network and database. J Clin Microbiol..

[20] Preston MD, Campino S, Assefa SA, Echeverry DF, Ocholla H, Amambua-Ngwa A (2014). A barcode of organellar genome polymorphisms identifies the geographic origin of *Plasmodium falciparum* strains. Nat Commun..

[21] Hikosaka K, Kita K, Tanabe K (2013). Diversity of mitochondrial genome structure in the phylum Apicomplexa. Mol Biochem Parasitol..

[22] da Silva AJ, Bornay-Llinares FJ, Moura IN, Slemenda SB, Tuttle JL, Pieniazek NJ (1999). Fast and reliable extraction of protozoan parasite DNA from fecal specimens. Mol Diagn..

[23] Qvarnstrom Y, Benedict T, Marcet PL, Wiegand RE, Herwaldt BL, da Silva AJ (2018). Molecular detection of *Cyclospora cayetanensis* in human stool specimens using UNEX-based DNA extraction and real-time PCR. Parasitology..

[24] Kumar S, Stecher G, Tamura K (2016). MEGA7: molecular evolutionary genetics analysis version 7.0 for bigger datasets. Mol Biol Evol..

[25] Guo Y, Wang Y, Wang X, Zhang L, Ortega Y, Feng Y (2019). Mitochondrial genome sequence variation as a useful marker for assessing genetic heterogeneity among *Cyclospora cayetanensis* isolates and source-tracking. Parasites Vectors..

[26] Su XZ, Wu Y, Sifri CD, Wellems TE (1996). Reduced extension temperatures required for PCR amplification of extremely A+T-rich DNA. Nucleic Acids Res..

[27] Pightling AW, Pettengill JB, Luo Y, Baugher JD, Rand H, Strain E (2018). Interpreting whole-genome sequence analyses of foodborne bacteria for regulatory applications and outbreak investigations. Front Microbiol..

[28] Pérez-Losada M, Arenas M, Castro-Nallar E (2018). Microbial sequence typing in the genomic era. Infect Genet Evol..

[29] Hoffmann M, Luo Y, Monday SR, Gonzalez-Escalona N, Ottesen AR, Muruvanda T (2016). Tracing origins of the *Salmonella* Bareilly strain causing a food-borne outbreak in the United States. J Infect Dis..

[30] Yang S, Hemarajata P, Hindler J, Li F, Adisetiyo H, Aldrovandi G (2017). Evolution and transmission of carbapenem-resistant *Klebsiella pneumoniae* expressing the blaOXA-232 gene during an institutional outbreak associated with endoscopic retrograde cholangiopancreatography. Clin Infect Dis..

[31] Davis GS, Waits K, Nordstrom L, Weaver B, Aziz M, Gauld L (2015). Intermingled *Klebsiella pneumoniae* populations between retail meats and human urinary tract infections. Clin Infect Dis..

[32] Guo Y, Roellig DM, Li N, Tang K, Frace M, Ortega Y (2016). Multilocus sequence typing tool for *Cyclospora cayetanensis*. Emerg Infect Dis..

[33] Guo Y, Li N, Ortega YR, Zhang L, Roellig DM, Feng Y (2018). Population genetic characterization of *Cyclospora cayetanensis* from discrete geographical regions. Exp Parasitol..

[34] Hofstetter JN, Nascimento FS, Park S, Casillas S, Herwaldt BL, Arrowood MJ (2019). Evaluation of multilocus sequence typing of *Cyclospora cayetanensis* based on microsatellite markers. Parasite..

[35] Barratt JLN, Park S, Nascimento FS, Hofstetter J, Plucinski M, Casillas S (2019). Genotyping genetically heterogeneous *Cyclospora cayetanensis* infections to complement epidemiological case linkage. Parasitology..

[36] Brown WM, George M, Wilson AC (1979). Rapid evolution of animal mitochondrial DNA. Proc Natl Acad Sci USA.

[37] Chikuni K, Mori Y, Tabata T, Saito M, Monma M, Kosugiyama M (1995). Molecular phylogeny based on the kappa-casein and cytochrome *b* sequences in the mammalian suborder Ruminantia. J Mol Evol..

[38] Lake JA (2015). Eukaryotic origins. Philos Trans R Soc Lond B Biol Sci..

[39] Budowle B, Allard MW, Wilson MR, Chakraborty R (2003). Forensics and mitochondrial DNA: applications, debates, and foundations. Annu Rev Genomics Hum Genet..

[40] Grzybowski T, Rogalla U (2012). Mitochondria in anthropology and forensic medicine. Adv Exp Med Biol..

[41] Forster P, Harding R, Torroni A, Bandelt HJ (1996). Origin and evolution of Native American mtDNA variation: a reappraisal. Am J Hum Genet..

[42] Denver DR, Morris K, Kewalramani A, Harris KE, Chow A, Estes S (2004). Abundance, distribution, and mutation rates of homopolymeric nucleotide runs in the genome of *Caenorhabditis elegans*. J Mol Evol..

[43] Denver DR, Morris K, Lynch M, Thomas WK (2004). High mutation rate and predominance of insertions in the *Caenorhabditis elegans* nuclear genome. Nature..

[44] Lynch M, Koskella B, Schaack S (2006). Mutation pressure and the evolution of organelle genomic architecture. Science..

[45] Melvin RG, Ballard JWO (2017). Cellular and population level processes influence the rate, accumulation and observed frequency of inherited and somatic mtDNA mutations. Mutagenesis..

[46] Nascimento FS, Barta JR, Whale J, Hofstetter JN, Casillas S, Barratt J (2019). Mitochondrial junction region as genotyping marker for *Cyclospora cayetanensis*. Emerg Infect Dis..

