# Supplementary material for: Molecular typing of Cyclospora cayetanensis in produce and clinical samples using targeted enrichment of complete mitochondrial genomes and next-generation sequencing
Source: Parasit Vectors. 2020 Mar 6;13:122. doi: 10.1186/s13071-020-3997-3 (PMC7060604; doi:10.1186/s13071-020-3997-3)
Supplement: Supplementary file 2 — Additional file 2: Table S2. List of mitochondrial genome assemblies from the present study. Table S3. Results of mapping and assembly of sequencing reads. Table S4. Variome matrix of mitochondria genome with SNP’s and InDels. [file 13071_2020_3997_MOESM2_ESM.docx]

**Additional file 2: Table S2** Mitochondrial genome assemblies from the present study

| **Biosample** | **Sample_Name** | **GenBank accession number** | **SRA accession** |
| --- | --- | --- | --- |
| SAMN10368222 | OH_14_CL_1 | MN260353 | SRX7365314 |
| SAMN10368223 | MA_14_CL_2 | MN260347 | SRX7366352 |
| SAMN10368224 | MA_14_CL_3 | MN316534 | SRX7366353 |
| SAMN10368225 | MA_14_CL_4 | MN260348 | SRX7366364 |
| SAMN10368226 | MT_14_CL_5 | MN260352 | SRX7366369 |
| SAMN10368227 | TX_14_CL_6 | MN260366 | SRX7366370 |
| SAMN10368228 | MA_14_CL_7 | MN260349 | SRX7366371 |
| SAMN10368230 | MA_14_CL_9 | MN260350 | SRX7366373 |
| SAMN10368231 | MA_14_CL_10 | MN260345 | SRX7366374 |
| SAMN10368232 | MA_14_CL_11 | MN260346 | SRX7366375 |
| SAMN10368234 | TX_14_CL_13 | MN260355 | SRX7366354 |
| SAMN10368235 | TX_14_CL_14 | MN260356 | SRX7366355 |
| SAMN10368236 | TX_14_CL_15 | MN260357 | SRX7366356 |
| SAMN10368237 | TX_14_CL_16 | MN260358 | SRX7366357 |
| SAMN10368238 | TX_14_CL_17 | MN260359 | SRX7366358 |
| SAMN10368239 | TX_14_CL_18 | MN260360 | SRX7366359 |
| SAMN10368240 | TX_14_CL_19 | MN260361 | SRX7366360 |
| SAMN10368241 | TX_14_CL_20 | MN260362 | SRX7366361 |
| SAMN10368242 | TX_14_CL_21 | MN260363 | SRX7366362 |
| SAMN10368243 | TX_14_CL_22 | MN260364 | SRX7366363 |
| SAMN10368244 | TX_14_CL_23 | MN260365 | SRX7366365 |
| SAMN10368245 | ME_14_CL_24 | MN316535 | SRX7366366 |
| SAMN10368246 | ME_14_CL_25 | MN260351 | SRX7366367 |
| SAMN10368247 | SC_14_CL_26 | MN260354 | SRX7366368 |

**Additional file 2: Table S3** Results of mapping and assembly of sequencing reads

| Sample name | Total reads | Percent mapped to reference | Average read length(bp) | Average Genome coverage |
| --- | --- | --- | --- | --- |
| TX_14_CL_17 | 1,061,928 | 88.43% | 236 | 35323X |
| TX_14_CL_21 | 421,888 | 68.96% | 228 | 10573 X |
| TX_14_CL_6 | 495,307 | 65.61% | 233 | 12068 X |
| TX_14_CL_22 | 2,458,275 | 98.29% | 224 | 86267 X |
| TX_14_CL_23 | 1,872,550 | 97.42% | 221 | 64258 X |
| MA_14_CL_8 | 1,500,664 | 88.39% | 223 | 47146 X |
| ME_14_CL_24 | 953,032 | 98.71% | 223 | 33437 X |
| ME_14_CL_25 | 1,454,727 | 98.88% | 220 | 50439 X |
| TX_14_CL_19 | 3,048,105 | 97.95% | 219 | 104215 X |
| TX_14_CL_18 | 1,453,070 | 97.18% | 227 | 51091 X |
| TX_14_CL_15 | 1,404,402 | 96.65% | 228 | 49326 X |
| TX_14_CL_14 | 1,590,973 | 98.47% | 223 | 55683 X |
| TX_14_CL_13 | 1,873,682 | 99.14% | 217 | 63695 X |
| MA_14_CL_11 | 1,255,778 | 97.47% | 215 | 41944 X |
| TX_14_CL_16 | 1,608,954 | 92.09% | 227 | 53609 X |
| MT_14_CL_5 | 1,074,826 | 96.87% | 223 | 37007 X |
| OH_14_CL_1 | 1,722,712 | 98.77% | 224 | 60478 X |
| MA_14_CL_3 | 2,574,590 | 95.31% | 223 | 87209 X |
| MA_14_CL_4 | 894,732 | 78.11% | 224 | 24952 X |
| MA_14_CL_10 | 2,401,753 | 96.54% | 214 | 79087 X |
| MA_14_CL_7 | 1,144,097 | 79.76% | 231 | 33598 X |
| MA_14_CL_9 | 364,798 | 66.72% | 231 | 8961 X |
| SC_14_CL_26 | 2,647,918 | 97.61% | 225 | 92691 X |
| TX_14_CL_20 | 1,129,282 | 97.01% | 222 | 38764 X |
| F200 (C5) | 441,112 | 86.31% | 225 | 13654 X |

**Additional file 2: Table S4: Variome matrix of mitochondria genome with SNPs and InDels**

**(Numbers 1-12 indicate the SNP positions according to KP231180, the reference genome; I=insertion; D=Deletion**

| **Study samples** | **KP231180** |
| --- | --- |
| TX_14_CL_19 | 2,3,4,6,D,11,12 |
| TX_14_CL_13 | 2,3,4,6,D,11,12 |
| TX_14_CL_23 | 2,3,4,6,D,11,12 |
| TX_14_CL_18 | 2,3,4,6,D,11,12 |
| TX_14_CL_14 | 2,3,4,6,D,11,12 |
| TX_14_CL_17 | 1,2,3,6,D, 11,12 |
|  |  |
| TX_14_CL_21 | 1,2,3,6,D |
| TX_14_CL_22 | 1,2,3,6,D |
| TX_14_CL_6 | 1,2,3,6,D |
|  |  |
| ME_14_CL_25 | 2,3,6,I |
| ME_14_CL_24 | 2,3,6,I |
| TX_14_CL_15 | 2,3,6,D |
|  |  |
| TX_14_CL_20 | 3,6 |
|  |  |
| MA_14_CL_10 | 3,4,6 |
|  |  |
| MA_14_CL_9 | 3,4,6,9,10,I |
| MA_14_CL_7 | 3,4,5,6,9,10,I |
| MA_14_CL_11 | 3,4,6,10, D |
| MA_14_CL_2 | 3,4,6,9,10,I,I |
| MA_14_CL_4 | 3,4,6,9,10,I |
| SC_14_CL_26 | 3,4,6,9,10,I |
|  |  |
| MA_14_CL_3 | 6,10 |
| OH_14_CL_1 | 6,8,10 |
| TX_14_CL_16 | 6,10,I |
|  |  |
| MG831587 | 6 |
| MT_14_CL_5 | 6,D |
| MG831586 | 6 |
| MG831588 | 6 |
